# Hypoxia Inducible Factor-1alpha (HIF-1A) plays different roles in Gallbladder Cancer and Normal Gallbladder Tissues

**DOI:** 10.7150/jca.46749

**Published:** 2021-01-01

**Authors:** Youliang Wu, Delong Meng, Yexiang You, Ruochuan Sun, Min Fu, Qiang Yan, Shangxin Zhang, Zheng Fang, Junjun Bao, Yongxiang Li

**Affiliations:** 1 Department of General Surgery, the First Affiliated Hospital of Anhui Medical University, Hefei 230022, People's Republic of China.; 2 Department of Molecular Biology, University of Texas Southwestern Medical Center, 6000 Harry Hines Blvd, Dallas, TX 75390, USA.

**Keywords:** gallbladder cancer, normal gallbladder tissue, HIF-1A, MVD, prognosis bio-marker

## Abstract

**Purpose:** Hypoxia-inducible factor-1alpha (HIF-1A) is a transcription factor that plays an “angiogenic switch” role especially under hypoxia microenvironment in solid tumor. However, the functions and clinical significance of HIF-1A in gallbladder cancer (GBC) are still controversial, and it has not been studied in normal gallbladder tissues. In this study, we sought to clarify the role of sub-cellular localization of HIF-1A expression in GBC and normal gallbladder tissues.

**Methods:** The expressions of HIF-1A and CD34 in 127 GBC and 47 normal gallbladder tissues were evaluated by immunohistochemistry. Cox's proportional hazards model analysis and Kaplan-Meier method analysis were used to assess the correlations between these factors and clinicopathological features and prognosis.

**Results:** HIF-1A was expressed in both cytoplasm and nucleus of GBC and normal control tissues, and was significantly correlated with microvessel density (MVD). GBC tissues with positive nuclear HIF-1A expression had higher MVD compared to that with positive cytoplasmic HIF-1A expression; however, in normal gallbladder tissues, samples with positive cytoplasmic HIF-1A had higher MVD compared to that with positive nuclear HIF-1A expression. Moreover, GBC with nuclear HIF-1A expression tended to be more poorly differentiated and had larger tumor size compared to that with cytoplasm HIF-1A expression. Furthermore, GBC patients with nuclear HIF-1A positive were significantly correlated with worse overall survival (OS) compared with cytoplasmic HIF-1A positive. Multivariate Cox regression analysis identified lymph node metastasis and nuclear HIF-1A expression to be independent prognostic parameter in GBC.

**Conclusions:** Our findings provide evidence for the first time that HIF-1A is expressed in normal gallbladder tissues. Nuclear HIF-1A and cytoplasm HIF-1A plays different roles in GBC and normal gallbladder tissues.

## Introduction

Gallbladder cancer (GBC) is a biliary tract malignant tumor and one of the most prevalent gastrointestinal cancers around the world 1. Although in recent years the diagnosis and treatment of GBC have improved continuously 2, only a minority of GBC patients are diagnosed at the early stages with favorable prognosis owing to lack of specific tumor bio-markers and highly with an aggressive behavior character 3. Therefore, it is imperative to find more valuable GBC molecular prognostic bio-markers and to study in-depth the molecular mechanism to guide for GBC treatment.

Hypoxia or anoxic environment is a common feature of many solids cancers, which is related to chemotherapy-resistant, invasion and metastasis, and malignant transformation 4-6. Hypoxia-inducible factor-1 (HIF-1) is a transcription factor composed of HIF-1α (HIF-1A) and HIF-1β subunits, which plays a pivotal role in the hypoxic adaptive response of tumors 7. Both subunits are ubiquitously expressed in all tissues except for peripheral blood, whereas the biological role of HIF-1 is determined by HIF-1A 8. Under normoxic conditions, HIF-1A protein is degraded by the proteasome signaling pathway 9, 10. However, under hypoxic conditions, HIF-1A becomes stable and degradation is prohibited, dimerizes with HIF-1β, and translocates from cytoplasm to nucleus, which induces the expression of a wide variety of target genes such as erythropoietin-1 (EPO-1), vascular endothelial growth factor (VEGF), glycolytic enzymes, and glucose transporter 1 (GLUT-1) to promote cell proliferation, angiogenesis, metastasis, and metabolic adaptation to hypoxia 8, 11, 12. HIF-1A has been intensively studied in malignant tumors. The relationship between the expression of HIF-1A and microvessel density (MVD) has mainly been investigated in many cancers, including GBC 13-15. In addition, HIF-1A is overexpressed in many cancers and its overexpression is often positively associated with poor prognosis 16-18. However, the prognostic value of HIF-1A in some cancers are still controversial, including GBC. In gastric cancer (GC), Sumiyoshi et al. found that overexpression of HIF-1A was associated with poor prognosis in GC patients 19; whereas Kolev et al. and Urano et al. found that overexpression of HIF-1A was not related to GC patient prognosis 20, 21. In GBC, Batmunkh et al. and Wu et al. found that the survival rate of HIF-1A positive staining patients was significantly lower than that of HIF-1A negative staining patients 22, 23; however, GIATROMANOLAKI et al. found no significant correlation between HIF-1A and survival rate 24. These controversy results were also observed in oral squamous cell carcinoma and ovarian cancer 25-28. HIF-1A expression in the cytoplasm or/and nucleus of cancer cells has been confirmed. Previous studies have shown that cytoplasm HIF-1A expression and nuclear HIF-1A expression play different roles in cancer progression 29, 30. As yet, no studies separately investigated the effect of cytoplasmic and nuclear HIF-1A expression on cancer patient progression. We infer that different localization of HIF-1A plays different roles in predicting clinical prognosis, which needs further dissection in GBC. Recently, HIF-1A was also found in some normal human tissue, such as retina, cartilage, epithelium of colon, dermal glands and hair follicles 8, 31, 32. In previous studies, HIF-1A was constantly negative in normal gallbladder tissue 22-24. However, our study found that HIF-1A was expressed in the cytoplasm or/and nucleus of normal human gallbladder tissues. This suggests that not only hypoxia but also other factors may contribute to HIF-1A activity and expression. We hypothesize that HIF-1A is constitutively expressed and may play a vital biological function in the normal gallbladder.

Until now, there is no consensus on the relationship between HIF-1A expression and GBC prognosis. Our group was the first to explore the relationship between sub-cellular localization of HIF-1A, prognosis and MVD of GBC. Furthermore, our results show for the first time that HIF-1A is also expressed in normal gallbladder tissues. To better understand the role of HIF-1A sub-cellular localization in GBC progression, this study aims to investigate the relationship between HIF-1A sub-cellular localization and clinicopathological features, MVD and prognosis in GBC.

## Materials and methods

### Ethics statement

This study obtained approval from the Institute Research Ethics Committee of the Armed Police Corps Hospital of Anhui and written informed consents were obtained by all patients involved.

### Patients and tissues specimens

In the present study, to construct tissue microarray (TMA) for immunohistochemical staining, a total of 127 formalin-fixed, paraffin-embedded GBC tissues and 47 randomly selected normal gallbladder tissues were collected between December 2004 and December 2014 from the Armed Police Corps Hospital of Anhui, Hefei, China. To get the complete patient's survival data, all patients were followed up every 2 months in the first 2 years and every 6 months in the following years. The complete follow-up date was updated to November 2016. Thorough histological features of these surgical specimens were confirmed using hematoxylin-eosin (H&E)-stained slides by experienced pathologists. Histological pathological TNM staging was assigned according to the seventh edition of TNM (tumor, lymph node, and metastasis) classification standards of American Joint Committee on Cancer (AJCC), which divides GBC into stage I, II, III, and IV from early to advanced stage: 83 cases of stage I/II and 44 cases of III/IV stage. In the 127 GBC patients, there were 36 males and 91 females. The histopathologic subtypes of the 127 GBC included 81 high/moderate and 46 low/undifferentiated. Detailed clinicopathological features of GBC were shown in **Tables 1-4.** As required, none of them received preoperative radiotherapy or chemotherapy.

### Immunohistochemistry (IHC)

HIF-1A and CD34 proteins were detected on TMA (4-um thick histological slides) by immunohistochemistry. The histological slides were baked at 63 °C for 1 h, and then deparaffinized in fresh xylene and dehydrated using a graded series of ethanol solutions. Antigen retrieval was performed in citrate buffer (0.01 M, PH 6.0) within a pressure cooker for 5 min at 120 °C. Subsequently, the slides were incubated with 3% hydrogen peroxide (H_2_O_2_) in methanol to quench the endogenous peroxidase activity, followed by incubation with 1% bovine serum albumin to block non-specific antigen-antibody reactivity. The sections were then incubated with primary antibodies (HIF-1A, 1:1000, abcam; CD34, 1:1000, ZSGB-BIO) at 4 °C overnight. After washing with PBS, the sections were incubated with secondary antibodies (horseradish peroxidase-labelled anti-rabbit IgG) at room temperature for 30 min to detect primary binding antibodies and washed with PBS. Finally, the sections were placed on an autostainer link instrument and proceed with staining. For negative control, the primary antibodies were replaced by normal rabbit IgG. Assessment of immunohistochemical staining scores was evaluated by two independent experienced pathologists without knowledge for this patient's pathologic information.

HIF-1A is expressed in cytoplasmic or/and nuclear. Cytoplasm and nuclear HIF-1A expression play different roles in cancer progression. Therefore, the cytoplasmic and the nuclear expression of HIF-1A protein were assessed separately and then combined in a grading system in our study. We classified HIF-1A intracellular immunoreactivity localization as nucleus-only, cytoplasm-only, nucleus and cytoplasm and negative-both. We divided these subgroups into nuclear-negative and -positive, cytoplasmic-negative and -positive. According to the intracellular immunoreactivity staining of GBC mucosa cells and normal mucosa cells, the percentage of positive mucosa cells stained was classified into four grades (0 point, no cells stained; 1 point, <25% positive cells; 2 points, 25-75% positive cells, 3 points, >75% positive cells); and the staining intensity of immunoreactivity was divided into four grades (0 point, negative; 1 point, weak intensity; 2 points, moderate intensity; 3 points, strong intensity). The immunoreactivity score (IRS) is derived by multiplying the two parameters. Specimens were attributed to two groups according to their IRS score: negative (-, IRS = 0 ~ 2), positive (+, IRS = 3 ~ 9).

Microvessel counting was performed to evaluate angiogenesis. Microvessels were assessed by immunostaining for CD34 with the accepted criteria according to Weidner et al. vessels with a clearly defined lumen, or well defined linear vessel shape rather than single endothelial cells, were used for microvessel counting 33. The selection sections were scanned at low magnification (100×) in the area of within the tumor or directly adjacent with the highest number of discrete microvessels (“hot spot”) under a light microscope (Leica, Germany). Then, microvessels were counted in five of the “hot spot” sections by the high magnification (200×) within an examination each area of 0.5 mm^2^. Final MVD counts were the mean of the vessel counts obtained in these five high magnification visual fields.

### Statistical analysis

All statistical analysis was carried out by SPSS 17.0 software (SPSS, Inc.Chicago, IL). Pearson χ2 test or Fisher's exact test were used to analyze correlations between nuclear HIF-1A expression level, cytoplasm HIF-1A expression level and clinicopathological features (including age, gender, tumor size, differentiation grade, depth of invasion, lymph node status and TNM stage, etc). The correlation between nuclear HIF-1A expression and cytoplasm HIF-1A expression was analyzed using the Spearman's rank test. Unpaired two-tailed t-test was used for testing relationship between HIF-1A expression and MVD counting. The survival time from the date of operation to death or the last follow-up was estimated in monthly terms. Overall survival (OS) was calculated by the Kaplan-Meier method, and the differences between two groups were tested by Log-Rank test. Multivariate Cox's proportional hazards model was only performed on the variables that are significantly associated with survival in univariate analysis to study their independence prognostic values. All *p*-value texts were two-sided. A* p*-value of less than 0.05 (*p* < 0.05) was considered statistically significant.

## Results

### Expression of HIF-1A in gallbladder cancer and normal gallbladder tissues

**Figure 1:**
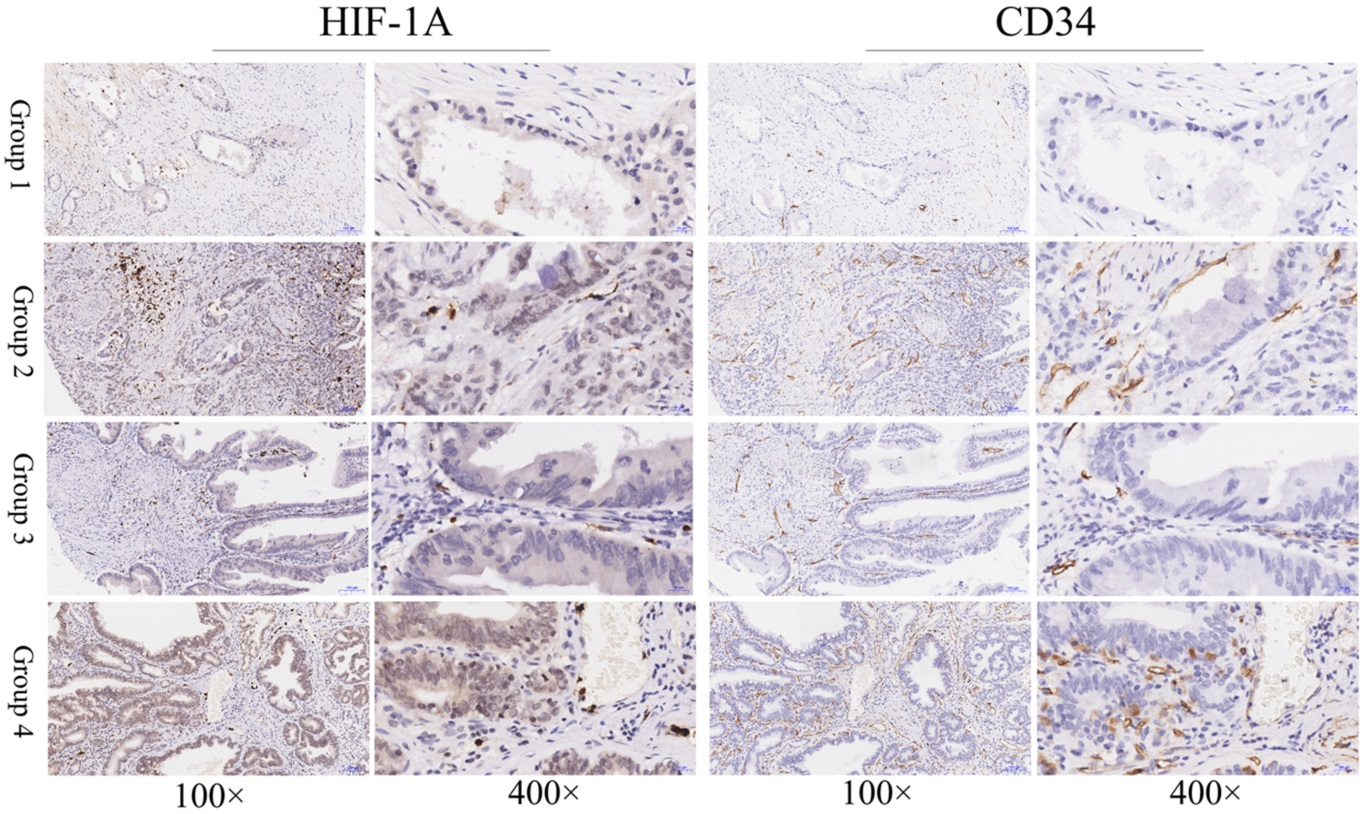
** Immunohistochemical staining of HIF-1A and CD34 in GBC tissues.** Representative images of HIF-1A and CD34 as followings: GBC tissues with cytoplasmic HIF-1A^-^/nuclear HIF-1A^-^ and low MVD (Group 1); GBC tissues with cytoplasmic HIF-1A^-^/nuclear HIF-1A^+^ and high MVD (Group 2); GBC tissues with cytoplasmic HIF-1A^+^/nuclear HIF-1A^-^ and low MVD (Group 3); GBC tissues with cytoplasmic HIF-1A^+^/nuclear HIF-1A^+^ and high MVD (Group 4). Magnification: 100× and 400×.

**Figure 2:**
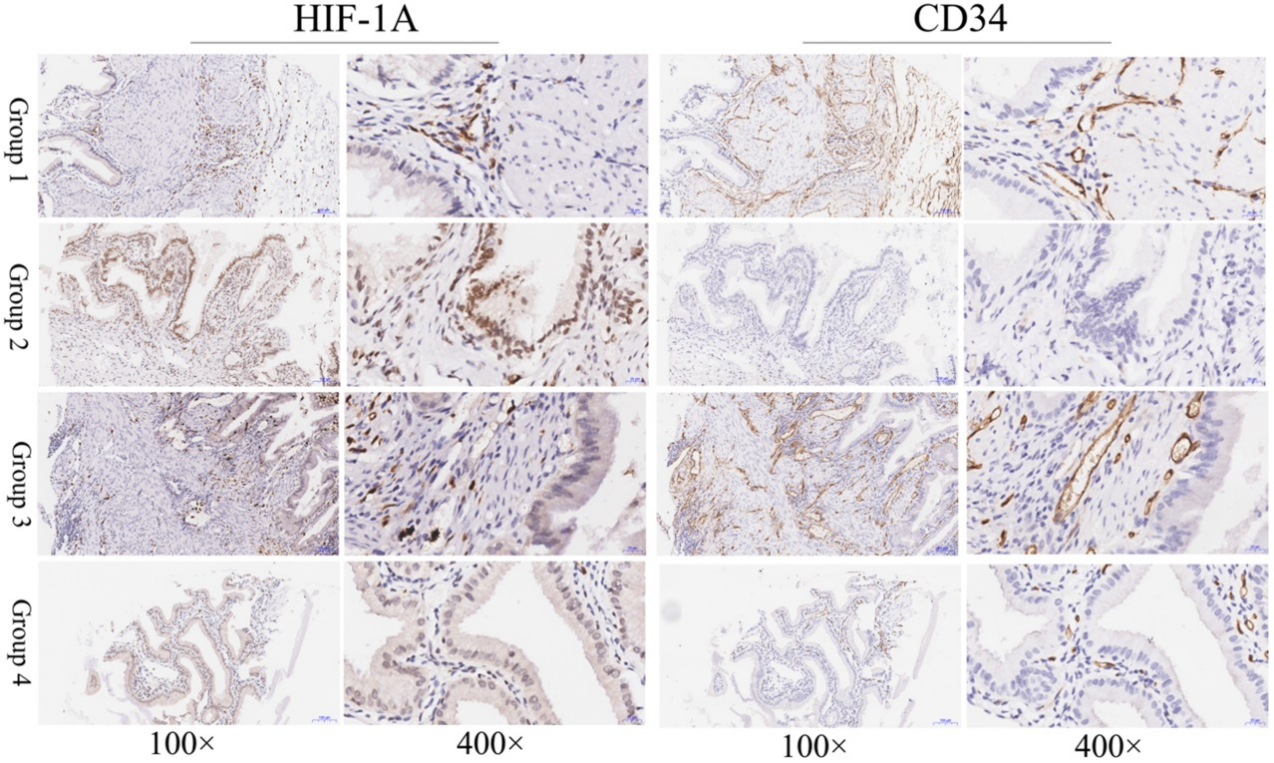
** Immunohistochemical staining of HIF-1A and CD34 in normal gallbladder tissues.** Representative images of HIF-1A and CD34 as followings: normal gallbladder tissues with cytoplasmic HIF-1A^-^/nuclear HIF-1A^-^ and middle MVD (Group 1); normal gallbladder tissues with cytoplasmic HIF-1A^-^/nuclear HIF-1A^+^ and low MVD (Group 2); normal gallbladder tissues with cytoplasmic HIF-1A^+^/nuclear HIF-1A^-^ and high MVD (Group 3); normal gallbladder tissues with cytoplasmic HIF-1A^+^/nuclear HIF-1A^+^ and middle MVD (Group 4). Magnification: 100× and 400×.

In this study, to determine the expression of HIF-1A in GBC and normal control, we assessed the expression level of HIF-1A in 127 GBC tissues and 47 normal control tissues on TMA by immunohistochemistry (**Figure 1 and Figure 2**). HIF-1A was expressed in the cytoplasm and/or nuclei of GBC and normal gallbladder cells, whereas at a varying extent. Although previous studies reported that HIF-1A protein was not expression in normal gallbladder cells, we found HIF-1A^+^ expression in 50.39% (64/127) of GBC samples, while in 87.23% (41/47) of normal gallbladder tissues (*p* < 0.001). Furthermore, higher percentage of overall HIF-1A expression in normal gallbladder tissues than GBC holds true for both cytoplasmic and nuclear expression of HIF-1A: 40.94% (52/127) of cytoplasmic HIF-1A^+^ in GBC samples versus 65.96% (31/47) in normal gallbladder samples (*p* = 0.003); and 21.26% (27/127) of nuclear HIF-1A^+^ in GBC samples versus 36.17% (17/47) in normal gallbladder samples (*p* = 0.045, **Tables 5 & 6**). In order to more precisely evaluate the difference of sub-cellular HIF-1A staining between the two groups, we divided the samples into 4 subgroups using the IRS criteria: group 1, cytoplasmic HIF-1A^-^/nuclear HIF-1A^-^; group 2, cytoplasmic HIF-1A^-^/nuclear HIF-1A^+^; group 3, cytoplasmic HIF-1A^+^/nuclear HIF-1A^-^; group 4, cytoplasmic HIF-1A^+^/nuclear HIF-1A^+^. With the above subgroup system, we found more of double negative (group 1) fraction in GBC samples (49.61%; 63/127) than in normal gallbladder samples (12.77%; 6/47; *p* < 0.001); less of nuclear only (group 2) fraction in GBCs (9.45%; 12/127) than in normal gallbladder tissues (21.28%; 10/47;* p* = 0.037); and similarly, less of cytoplasm only (group 3) fraction in GBCs (29.13%; 37/127) than in normal gallbladder tissues (50.06%; 24/47; *p* = 0.007). However, we didn't observe difference in the distribution of double positive samples (group 4), which accounts for 11.81% (15/127) in GBC samples and 14.89% (7/47) in normal gallbladder samples (*p* = 0.587).

**Figure 3:**
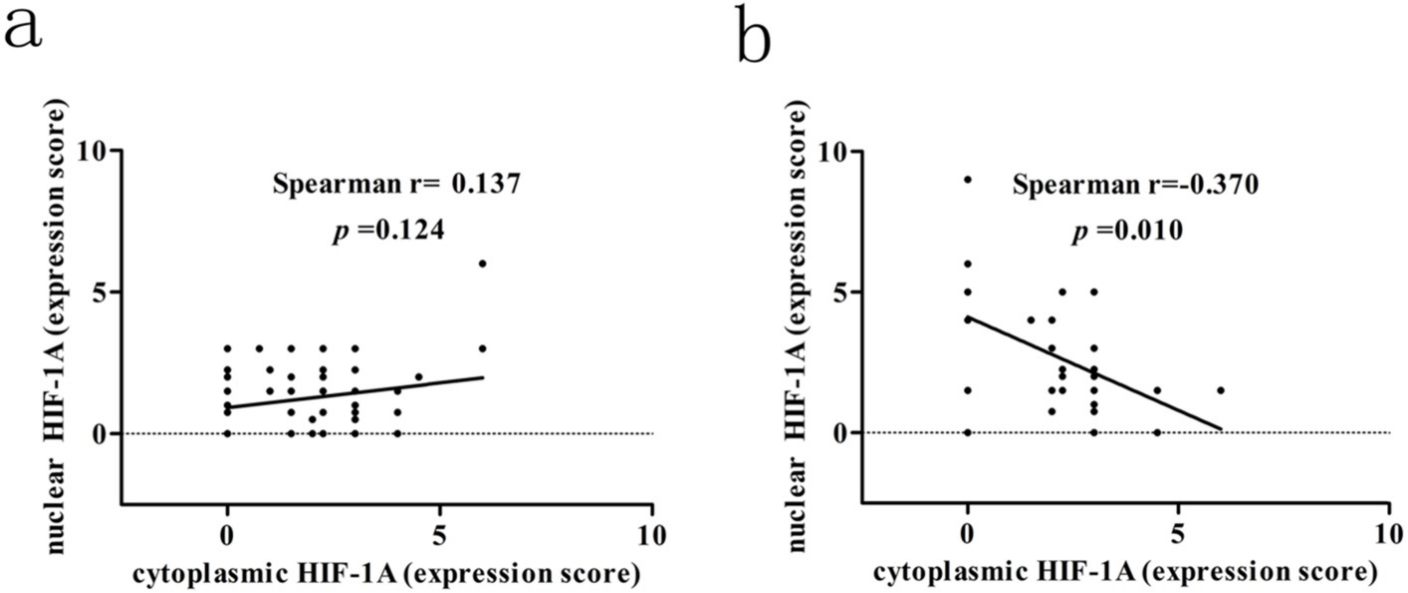
** Correlation between cytoplasmic HIF-1A and nuclear HIF-1A expression levels in GBC and normal gallbladder tissues. a.** The correlation between cytoplasmic HIF-1A and nuclear HIF-1A expression levels in GBC tissues. **b.** The correlation between cytoplasmic HIF-1A and nuclear HIF-1A expression levels in normal gallbladder tissues.

We next assessed the association between cytoplasmic and nuclear HIF-1A expression in GBC and normal gallbladder tissues, respectively. While we failed to observe any association between cytoplasmic and nuclear HIF-1A expression in GBC (r = 0.137, *p* = 0.124, **Figure 3a**), we discovered that in normal gallbladder tissues cytoplasmic HIF-1A expression was negatively correlated with nuclear HIF-1A expression (r = -0.370, *p* = 0.010, **Figure 3b**). Taken together, we found that HIF-1A is expressed in normal control tissues, at a degree comparable to or even higher than in GBCs, and interestingly, correlations between the sub-cellular distributions of HIF-1A differ in GBCs and normal gallbladder tissues.

### Correlation between MVD and HIF-1A expression

**Figure 4:**
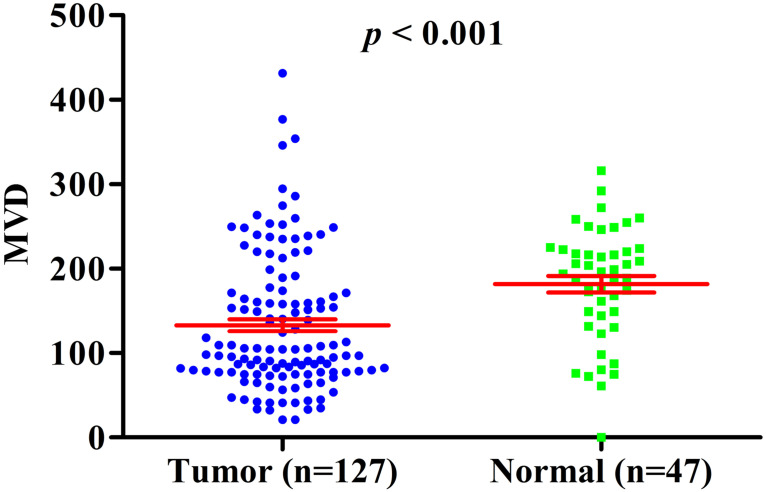
MVD counting in GBC compared with normal gallbladder tissues.

**Figure 5:**
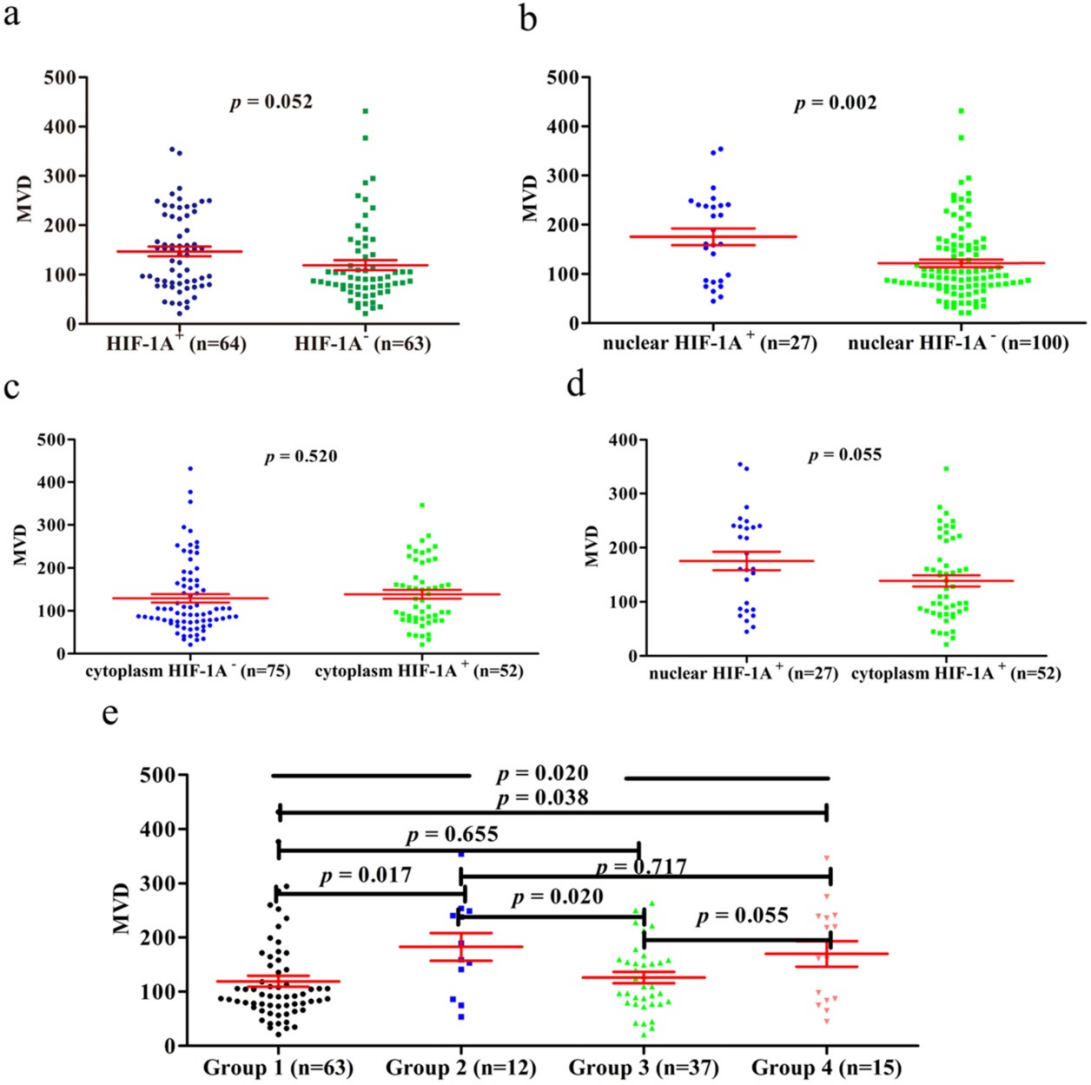
** MVD counting of GBC tissues. a.** MVD counting in GBC tissues with HIF-1A^+^ and HIF-1A^-^. **b.** MVD counting in GBC tissues with nuclear HIF-1A^+^ and nuclear HIF-1A^-^. **c.** MVD counting in GBC tissues with cytoplasmic HIF-1A^+^ and cytoplasmic HIF-1A^-^. **d.** MVD counting in GBC tissues with nuclear HIF-1A^+^ and cytoplasmic HIF-1A^+^. **e.** MVD counting in GBC tissues with subgroups of cytoplasmic HIF-1A/nuclear HIF-1A.

**Figure 6:**
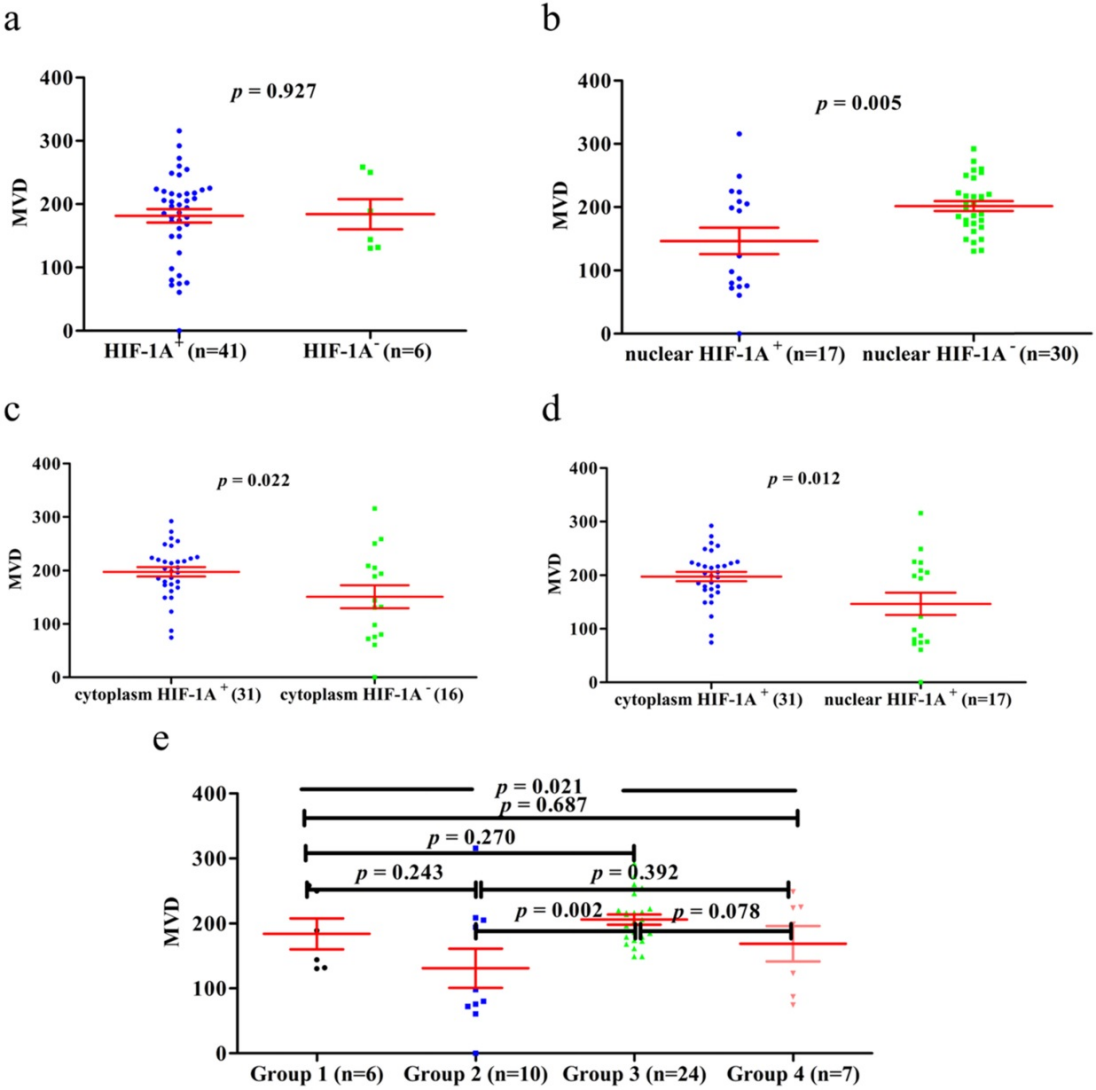
** MVD counting of normal gallbladder tissues. a.** MVD counting in normal gallbladder tissues with HIF-1A^+^ and HIF-1A^-^. **b.** MVD counting in normal gallbladder tissues with nuclear HIF-1A^+^ and nuclear HIF-1A^-^. **c.** MVD counting in normal gallbladder tissues with cytoplasmic HIF-1A^+^ and cytoplasmic HIF-1A^-^. **d.** MVD counting in normal gallbladder tissues with nuclear HIF-1A^+^ and cytoplasmic HIF-1A^+^. **e.** MVD counting in normal gallbladder tissues with subgroups of cytoplasmic HIF-1A/nuclear HIF-1A.

Previous studies have shown that HIF-1A may regulate tumor angiogenesis, but in GBC and normal gallbladder controls, the relationship between cytoplasm HIF-1A expression, nuclear HIF-1A expression and MVD has not been studied. Therefore, we checked whether there were any correlations between cytoplasm HIF-1A expression, nuclear HIF-1A expression and MVD in GBC and normal gallbladder tissues. MVD was evaluated by counting CD34 immunohistochemistry staining. Generally, the average MVD counting in GBC (133 ± 7.192) was significantly lower than that in normal gallbladder tissues (181 ± 9.741, *p* < 0.001, **Figure 4**). This result indicates that not all tumor tissues have higher MVD than normal controls, at least in GBC. Indeed, this phenomenon was also observed in another type of tumor we studied (data not shown). Furthermore, we compared MVD in HIF-1A positive or negative samples and found that in GBC samples, the MVD in HIF-1A^+^ group (147 ± 9.828) were higher than that in HIF-1A^-^ group (119 ± 10.290, *p* = 0.052, **Figure 5a**), although the difference did not reach statistical significance. Taking sub-cellular localization of HIF-1A into account, we found that in GBC samples, MVD in nuclear HIF-1A^+^ group (175 ± 17.020) were higher than that in nuclear HIF-1A^-^ group (121 ± 7.540, *p* = 0.002, **Figure 5b**), whereas we didn't find any difference of MVD between cytoplasm HIF-1A^+^ group (138 ± 10.360) and cytoplasm HIF-1A^-^ group (129 ± 9.867, *p* = 0.520, **Figure 5c**). Moreover, we found that the MVD in nuclear HIF-1A^+^ group (175 ± 17.020) were higher than that in cytoplasm HIF-1A^+^ group (138 ± 10.360, *p* = 0.055, **Figure 5d**), although the difference did not reach statistical significance. In normal gallbladder tissues, we didn't find any difference of MVD between HIF-1A^+^ group (181 ± 10.710) and HIF-1A^-^ group (184 ± 23.880, *p* = 0.927, **Figure 6a**). MVD in nuclear HIF-1A^+^ group (146 ± 20.910) were lower than that in nuclear HIF-1A^-^ group (201 ± 7.834, *p* = 0.005, **Figure 6b**), and MVD in cytoplasm HIF-1A^+^ group (197±8.836) were higher than that in cytoplasm HIF-1A^-^ group (151 ± 21.370, *p* = 0.022, **Figure 6c**). Moreover, we found that the MVD in nuclear HIF-1A^+^ group (146 ± 20.910) were lower than that in cytoplasm HIF-1A^+^ group (197 ± 8.836, *p* = 0.012, **Figure 6d**). From the above results, it is known that nuclear HIF-1A and cytoplasm HIF-1A play different roles in angiogenesis of GBC and normal gallbladder tissues.

We then proceeded to study the correlation between MVD and different combinations of cytoplasm HIF-1A expression and nuclear HIF-1A expression in GBC and normal gallbladder tissues. In GBC, cytoplasm HIF-1A^-^/nuclear HIF-1A^+^ subgroup (182 ± 25.53) and cytoplasm HIF-1A^+^/nuclear HIF-1A^+^ subgroup (169 ± 23.53) had higher MVD than other subgroups (**Figure 5e**). In normal gallbladder tissues, cytoplasm HIF-1A^+^/nuclear HIF-1A^-^ subgroup (206 ± 7.845) and cytoplasm HIF-1A^-^/nuclear HIF-1A^-^ subgroup (184 ± 23.88) had higher MVD than other subgroups (**Figure 6e**). In summary, our results suggest that nuclear HIF-1A expression is more likely to be positively correlated with MVD in GBC while negatively correlated with MVD in normal gallbladder tissues.

### Relationship between HIF-1A expression and clinicopathological parameters of GBC

To further analyze the clinical significance of HIF-1A expression in GBC, we examined the relationship between HIF-1A expression and clinicopathological features (including gender, age, tumor size, histological differentiation grade, lymph node metastasis, depth of invasion and TNM stage, etc) in GBC patients. TNM tumor staging can be divided into early stage (I/II) and advanced stage (III/IV); histological differentiation grade was divided into high/moderate and low/undifferentiated; lymph node staging was divided into positive lymph node metastasis (yes) and negative lymph node metastasis (no). As shown in **Table 1**, we did not find any significant association between HIF-1A overall expression and clinicopathological features.

However, nuclear or cytoplasmic expression of HIF-1A appears to be associated with some clinical features including tumor size and differentiation (**Tables 2 & 3**). When further comparing samples with nuclear or cytoplasmic expression of HIF-1A (**Table 4**), we found that samples with only nuclear HIF-1A expression tended to be more poorly differentiated and have larger tumor size compared to that with only cytoplasm HIF-1A expression, although the difference did not reach statistical significance, probably due to the small sample size. Taken together, our findings indicate that nuclear HIF-1A^+^ is more prone to malignancy.

### Survival analysis

**Figure 7:**
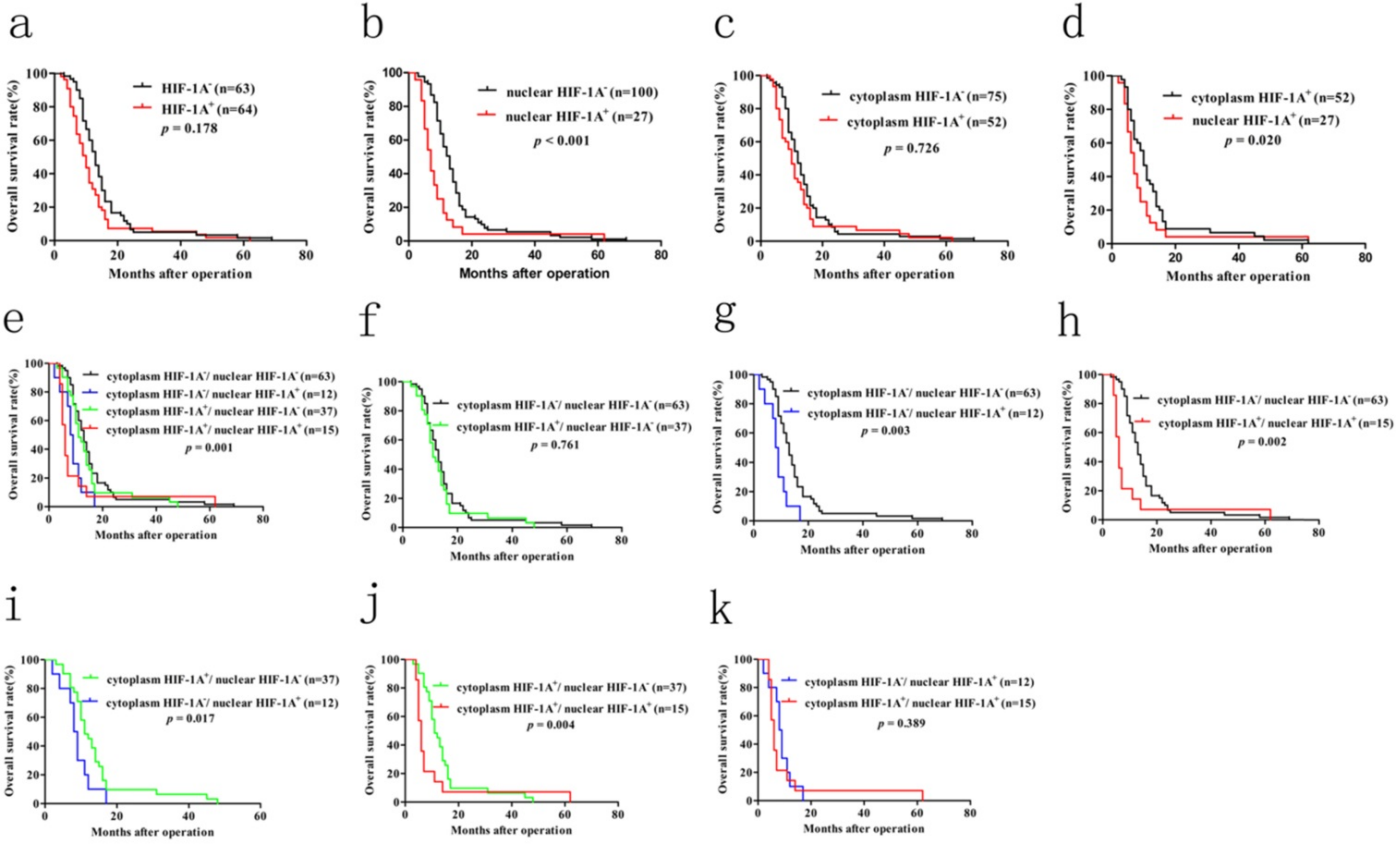
** Kaplan-Meier survival analysis with Log-Rank test for the OS of 127 GBC patients. a.** The OS of GBC patients with HIF-1A^+^ and HIF-1A^+^.** b.** The OS of GBC patients with nuclear HIF-1A^+^ and nuclear HIF-1A^-^. **c.** The OS of GBC patients with cytoplasmic HIF-1A^+^ and cytoplasmic HIF-1A^-^. **d.** The OS of GBC patients with cytoplasmic HIF-1A^+^ and nuclear HIF-1A^+^. **e, f, g, h, i, j, k.** The OS of GBC patients with subgroups stratified to cytoplasmic HIF-1A/nuclear HIF-1A expressions.

Previous studies have shown that overexpression of HIF-1A in many cancer patients is associated with poor prognosis, including GBC. However, until now there is still no consensus on the relationship between HIF-1A expression and GBC prognosis. In order to determine the prognostic values of HIF-1A expression for GBC, we used Kaplan-Meier method and log-rank test to analyze the survival rate. Kaplan-Meier analysis of GBC patients with HIF-1A^+^ [median 10 months, mean 13.281 ± 1.612 months] showed a trend of shorter overall survival (OS) than that of HIF-1A^-^ patients [median 13 months, mean 15.587 ± 1.400 months, *p* = 0.178, **Figure 7a**]. GBC patients with positive nuclear HIF-1A (HIF-1A^+^) had worse OS [median 7 months, mean 9.370 ± 2.216 months] than those with negative nuclear HIF-1A (HIF-1A^-^) [median 13 months, mean 15.790 ± 1.200 months, *p* < 0.001, **Figure 7b**]. In contrast, GBC patients with cytoplasm HIF-1A^+^ had no significant difference in OS [median 10 months, mean 14.423 ± 1.936 months] than those with cytoplasm HIF-1A^-^ [median 12 months, mean 14.427 ± 1.227 months, *p* = 0.726, **Figure 7c**]. When comparing GBC patients with nuclear or cytoplasmic expression of HIF-1A, we found that patients with nuclear HIF-1A^+^ had worse OS [median 7 months, mean 9.370 ± 2.216 months] than those with cytoplasm HIF-1A^+^ [median 10 months, mean 14.423 ± 1.936 months, *p* = 0.020, **Figure 7d**].

In order to make a distinction the clinical significance of cytoplasm HIF-1A and nuclear HIF-1A, we examined the relationship between the different combinations of cytoplasm HIF-1A and nuclear HIF-1A and OS. Kaplan-Meier analysis of GBC patients with cytoplasm HIF-1A^-^/nuclear HIF-1A^+^ or cytoplasm HIF-1A^+^/nuclear HIF-1A^+^ subgroups showed a significantly worse overall survival (OS) than that of other subgroups (**Figure 7e, 7g-j**). There was no significant difference between the patients with cytoplasm HIF-1A^-^/nuclear HIF-1A^+^ [median 8.5 months, mean 8.333 ± 1.157 months] and cytoplasm HIF-1A^+^/nuclear HIF-1A^+^ subgroups [median 6 months, mean 10.200 ± 3.763 months, *p* = 0.398, **Figure 7k**] on OS. This phenomenon was also found between cytoplasm HIF-1A^-^/nuclear HIF-1A^-^ subgroup [median 13 months, mean 15.587 ± 1.400 months] and cytoplasm HIF-1A^+^/nuclear HIF-1A^-^ subgroup [median 12 months, mean 16.135 ± 2.224 months, *p* = 0.761, **Figure 7f**]. In conclusion, these results suggest that nuclear HIF-1A expression is significantly associated with poor clinical prognosis of GBC, regardless of the status cytoplasm HIF-1A. Furthermore, in order to find independent prognostic factors, we carried out uni- and multivariate Cox's proportional hazards regression model to examine the prognostic significance of HIF-1A expression and other clinical parameters.

In univariate analysis, we found that histological differentiation grade (*p* = 0.003), depth of invasion (*p* = 0.016), lymph node metastasis (*p* = 0.002), TNM stages (*p* = 0.011), nuclear HIF-1A expression (*p* **<** 0.001) were statistically significant factors for OS (**Table 7**). We further performed multivariate survival analyses combining these statistically significant factors. We found lymph node metastasis [*p* = 0.032, HR (hazard ratio) = 2.435, 95% CI: 1.078-5.502] and nuclear HIF-1A expression [*p* =0.002, HR (hazard ratio) = 2.129, 95% CI: 1.308-3.466] as independent prognostic factors for OS in GBC patients (**Table 7**). In sum, our results further suggest that nuclear HIF-1A expression, rather than cytoplasm HIF-1A expression, is significantly associated with poor clinical outcomes of GBC, and could serve as an independent prognostic factor for GBC.

## Discussion

HIF-1A is a transcription factor expressed in the cytoplasm or/and nucleus of many cancers, but rarely detected in the normal control tissues. Recently, HIF-1A has also been found in some normal human tissues and participates in important physiological functions 8, 31, 32, 34. Giles et al. reported that HIF-1A expression seems to play a protective role in the intestinal epithelium in genetic models 32; Pfander et al. reported that HIF-1A was a highly conserved transcription factor, which plays a key role in energy generation, cell survival and matrix synthesis of articular and growth plate chondrocytes 34. In our study, we first studied the sub-cellular expression of HIF-1A separately. Also, we reported for the first time that HIF-1A was expressed in normal gallbladder tissues, and both cytoplasm and nucleus were higher than GBC. This result is different with previous reports, but consistent with THE HUMAN PROTEIN ATLAS database. THE HUMAN PROTEIN ATLAS database shows that HIF-1A is highly expressed in gallbladder tissue. Batmunkh et al. and Wu et al. reported that HIF-1A was not expressed in normal gallbladder tissues 22, 23. This discrepancy may be related to the quality and quantity of samples, the classification and staging of tumors, the population, or hypoxia-independent mechanism - such as VHL inactivation, proinflammatory mediators, etc. In addition, our findings are consistent with those of Hughes et al., Rosenberger et al. and Giles et al., whose findings confirmed HIF-1A expression in normal human tissues, not just in tumors 8, 31, 32. Therefore, individual/local variation in oxygen levels, VHL inactivation or proinflammatory mediators in the human GBC and normal gallbladder tissue may affect the nuclear and cytoplasmic HIF-1A levels. Thus far, only Giatromanolaki et al. mentioned that nuclear HIF-1A expression was accompanied with moderate/strong cytoplasmic reactivity, but there was no data to prove it 24. In our results, we discovered that cytoplasmic HIF-1A expression were negatively correlated with nuclear HIF-1A expression in normal gallbladder tissues using the Spearman's rank test; however, we didn't observe any relationship between cytoplasmic HIF-1A expression and nuclear HIF-1A expression in GBC. This result indicated that cytoplasmic HIF-1A expression and nuclear HIF-1A expression may play independent function and have different expression regulation mechanisms between in GBC and normal gallbladder tissues. Thus, our findings agreed that HIF-1A is constitutively expressed in normal gallbladder tissue and may play an important role in physiology, which requires further study.

Previous studies have reported that nuclear HIF-1A expression plays an important role in biological function. Nuclear import of HIF-1A as a transcription factor is a hallmark event in HIF-1 dependant hundreds of target gene activation, such as angiogenic gene including VEGF-A, Flt-1, ang-1, MMP-2 and MMP-9 which promotes tumour angiogenesis, metastasis and clinical prognosis 29. To date, there is no evidence to support the cytoplasmic function of HIF-1A, which may be due to the instability of cytoplasmic HIF-1A and has not been further studied. In our study, we found that the effects of HIF-1A expressed in nucleus and cytoplasm were different on angiogenesis and clinical prognosis. For angiogenesis, heterogeneous correlation between HIF-1A expression and MVD was observed in GBC and normal gallbladder tissues. In GBC, we found MVD in cytoplasm HIF-1A^-^/nuclear HIF-1A^+^ subgroup or cytoplasm HIF-1A^+^/nuclear HIF-1A^+^ subgroup was significant higher compared with other subgroups. There was no significance difference in MVD between cytoplasm HIF-1A^-^/nuclear HIF-1A^+^ subgroup and cytoplasm HIF-1A^+^/nuclear HIF-1A^+^ subgroup. We also observed no significance difference in MVD between cytoplasm HIF-1A^+^/nuclear HIF-1A^-^ subgroup and cytoplasm HIF-1A^-^/nuclear HIF-1A^-^ subgroup. These findings were consistent with previous reports in tumors. MVD was higher in nuclear HIF-1A expression, and nuclear HIF-1A determined the functional activity of the HIF-1A complex 35, 36. In normal gallbladder tissues, we found that MVD in cytoplasm HIF-1A^+^/nuclear HIF-1A^-^ subgroup was significantly higher than that of cytoplasm HIF-1A^-^/nuclear HIF-1A^+^ subgroup. Nuclear HIF-1A expression seems to be inversely correlated with MVD, while cytoplasm HIF-1A seems to be positively correlated with MVD, suggesting that nuclear and cytoplasmic HIF-1A play different roles in the angiogenesis of normal gallbladder tissue and GBC. This finding is extraordinary discrepancy from that found in GBC. Although very little is known about the functions of cytoplasmic HIF-1A, it is expected that cytoplasmic HIF-1A has specialized roles in angiogenesis of GBC and normal gallbladder tissue. This discrepancy may be due to the various functions of cytoplasm HIF-1A and nuclear HIF-1A in GBC and normal gallbladder tissues, or to the more complex mechanism of angiogenesis in normal gallbladder tissues. In addition, we found that MVD in the normal control group was significantly higher than that in GBC. This finding was extraordinary discrepancy from previous studies. In GBC, Stancu et al. and Niu et al. reported that MVD in cancer tissues were significantly higher than that in normal gallbladder tissues 37, 38. In their study, there were very few cancer samples and control samples, which may be one of the reasons. The similar findings were found in other tumors, LI et al., Gao et al. and Li et al. reported that MVD in oral cancer, breast cancer and gastric cancer were significantly higher than that in normal tissues, separately 39-41. This phenomenon may be related to the heterogeneity of tumors. However, we were more inclined to believe that tumors like hypoxic microenvironment. Hypoxia can promote tumorigenesis and progression through a variety of mechanisms, including epithelial - mesenchymal transformation (EMT), triggering angiogenesis and affecting angiogenesis mimicry, remodeling extracellular matrix, promoting tumor immune escape and anaerobic glycolysis, maintaining the existence of cancer stem cells, inhibiting aging, promoting the proliferation of cancer cells 42-45. Therefore, even the tumor hypoxia microenvironment promotes the production of MVD, there will not be as much MVD as normal tissue to provide more oxygen, because tumors prefer the hypoxic environment. This gives us reason to believe that the MVD in GBC is lower than that of the normal gallbladder tissues.

Regarding HIF-1A's effect on prognosis, as yet, no studies separately investigated the effect of cytoplasmic and nuclear HIF-1A expression on GBC progression. We examined the relationship between HIF-1A expression and clinicopathological features. We found that nuclear HIF-1A expression more tended to be poorly differentiated and large tumor size compared with cytoplasm HIF-1A expression, which supported that cytoplasm HIF-1A and nuclear HIF-1A played different roles in cancer progression. Several independent studies reported that increased HIF-1A expression was associated with poor prognosis in many cancers 16, 46, but the prognostic value of HIF-1A expression in GBC is still controversial 22-24, however, we did not study the relationship between cytoplasmic HIF-1A expression and nuclear HIF-1A expression and GBC prognosis separately. We found GBC patients with HIF-1A^+^ showed a trend short OS than that of HIF-1A^-^ patients, but it was not reach statistically significant. This finding agrees with the reports of GIATROMANOLAKI et al. 24. The data suggested that HIF-1A is involved in the tumorigenesis and progression of GBC, but is only a weak prognostic factor. We studied the relationship between cytoplasmic HIF-1A expression and nuclear HIF-1A expression and GBC prognosis separately. We found that GBC patients with cytoplasm HIF-1A^-^/ nuclear HIF-1A^+^ or cytoplasm HIF-1A^+^/nuclear HIF-1A^+^ subgroup showed a significantly worse OS than that of other subgroups. This finding was consistent with the report by Batmunkh et al. that HIF-1A may be a molecular prognostic indicator for GBC patients 22. This may be related to nuclear HIF-1A (as a highly conserved transcription factor), which regulates the expression of multiple target genes that are closely associated with tumor growth, angiogenesis, metastasis, venous invasion and chemotherapy resistance, thus promoting the tumor progression of GBC 47. Since neither of these studies investigated the prognosis value of GBC with cytoplasm and nuclear HIF-1A expression separately, it was possible that cytoplasm masked the role of HIF-1A in the nucleus as shown in this study. Moreover, our multivariate Cox analysis further demonstrated that nuclear HIF-1A could be an independent prognostic factor for GBC. These results indicated that nuclear HIF-1A expression was a more reliable predictor of GBC prognosis compared with cytoplasm HIF-1A expression, so we should separately investigated the effect of cytoplasmic and nuclear HIF-1A expression on GBC progression.

In conclusion, this study was the first comprehensive analysis of the expression and role of HIF-1A in GBC and normal gallbladder tissues. We found that HIF-1A protein was frequently expressed in GBC and normal gallbladder tissues, and both cytoplasmic and nuclear HIF-1A expression were higher in normal gallbladder tissues than in GBC, which provided evidence for the constitutive expression of HIF-1A in the normal gallbladder tissues. In addition, we showed that nuclear HIF-1A expression is more likely to be positively correlated with MVD in GBC while negatively correlated with MVD in normal gallbladder tissues, which indicated that nuclear HIF-1A plays different roles in angiogenesis between normal gallbladder tissues and GBC, and the specific mechanism needs further study. Furthermore, we implied that nuclear HIF-1A could be an independent prognostic factor for GBC and act as a more reliable predictor of GBC prognosis compared with cytoplasmic HIF-1A. This finding implicated that nuclear HIF-1A could act as a novel therapeutic molecular target for GBC. From a future perspective, biological agents targeting nuclear HIF-1A might be more effective in treating GBC.

## Figures and Tables

**Table 1:** Relationship between HIF-1A expression and clinicopathological variables (n=127)

Clinicopathological variables	Total	HIF-1A expression		*p* value
		positive (64)	negative (63)	
**Gender**				0.216
Male	36	15	21	
Female	91	49	42	
**Age (y)**				0.129
<68	59	34	25	
≥68	68	30	38	
**Tumor size (cm)**				0.786
<2	67	33	34	
≥2	60	31	29	
**Differentiation**				0.421
High/moderate	81	43	38	
Low/undifferentiated	46	21	25	
**Depth of invasion**				0.198
T1/T2	39	23	16	
T3/T4	88	41	47	
**Lymph node metastasis**				0.18
Yes	51	22	29	
No	76	42	34	
**TNM**				0.418
I/II	83	44	39	
III/IV	44	20	24	
**Gallstones**				0.317
Yes	84	45	39	
No	43	19	24	
**AFP (ug/L)**				1
<20	122	61	61	
≥20	5	3	2	
**CEA (ng/ML)**				0.299
<5	100	48	52	
≥5	27	16	11	
**CA199 (U/ML)**				0.948
<37	83	42	41	
≥37	44	22	22	

Note: TNM, tumor-node-metastasis; AFP, alpha fetoprotein; CEA, carcino-embryonic antigen; CA199, carbohydrate antigen 199; *p*<0.05 was defined statistically significant.

**Table 2:** Relationship between nuclear HIF-1A expression and clinicopathological variables (n=127)

Clinicopathological variables	Total	Nuclear HIF-1A expression		*p* value
		positive (27)	negative (100)	
**Gender**				0.202
Male	36	5	31	
Female	91	22	69	
**Age (y)**				0.843
<68	59	13	46	
≥68	68	14	54	
**Tumor size (cm)**				**0.065**
<2	67	10	57	
≥2	60	17	43	
**Differentiation**				0.316
High/moderate	81	15	66	
Low/undifferentiated	46	12	34	
**Depth of invasion**				0.422
T1/T2	39	10	29	
T3/T4	88	17	71	
**Lymph node metastasis**				0.162
Yes	51	14	37	
No	76	13	63	
**TNM**				0.228
I/II	83	15	68	
III/IV	44	12	32	
**Gallstones**				0.948
Yes	84	18	66	
No	43	9	34	
**AFP (ug/L)**				0.626
<20	122	25	97	
≥20	5	2	3	
**CEA (ng/ML)**				0.231
<5	100	19	81	
≥5	27	8	19	
**CA199 (U/ML)**				0.453
<37	83	16	67	
≥37	44	11	33	

**Table 3:** Relationship between cytoplasmic HIF-1A expression and clinicopathological variables (n=127)

Clinicopathological variables	Total	Cytoplasmic HIF-1A expression		*p* value
		positive (52)	negative (75)	
**Gender**				0.486
Male	36	13	23	
Female	91	39	52	
**Age (y)**				0.304
<68	59	27	32	
≥68	68	25	43	
**Tumor size (cm)**				0.571
<2	67	29	38	
≥2	60	23	37	
**Differentiation**				**0.069**
High/moderate	81	38	43	
Low/undifferentiated	46	14	32	
**Depth of invasion**				0.687
T1/T2	39	17	22	
T3/T4	88	35	53	
**Lymph node metastasis**				0.072
Yes	51	16	35	
No	76	36	40	
**TNM**				0.128
I/II	83	38	45	
III/IV	44	14	30	
**Gallstones**				0.169
Yes	84	38	46	
No	43	14	29	
**AFP (ug/L)**				0.674
<20	122	49	73	
≥20	5	3	2	
**CEA (ng/ML)**				0.981
<5	100	41	59	
≥5	27	11	16	
**CA199 (U/ML)**				0.700
<37	83	35	48	
≥37	44	17	27	

**Table 4:** Relationship between cytoplasmic HIF-1A^-^/nuclear HIF-1A^+^, cytoplasmic HIF-1A^+^/nuclear HIF-1A^-^ expression and clinicopathological variables (n=49)

Clinicopathological variables	Total	cytoplasmic HIF-1A^-^/ nuclear HIF-1A^+^ (12)	cytoplasmic HIF-1A^+^/ nuclear HIF-1A^-^ (37)	*p* value
**Gender**				0.735
Male	12	2	10	
Female	37	10	27	
**Age (y)**				0.924
<68	28	7	21	
≥68	21	5	16	
**Tumor size (cm)**				**0.081**
<2	27	4	23	
≥2	22	8	14	
**Differentiation**				**0.067**
High/moderate	33	5	28	
Low/undifferentiated	16	7	9	
**Depth of invasion**				0.564
T1/T2	19	6	13	
T3/T4	30	6	24	
**Lymph node metastasis**				0.128
Yes	14	6	8	
No	35	6	29	
**TNM**				0.128
I/II	35	6	29	
III/IV	14	6	8	
**Gallstones**				0.551
Yes	34	7	27	
No	15	5	10	
**AFP (ug/L)**				1
<20	48	12	36	
≥20	1	0	1	
**CEA (ng/ML)**				0.322
<5	36	7	29	
≥5	13	5	8	
**CA199 (U/ML)**				0.68
<37	33	7	26	
≥37	16	5	11	

**Table 5:** The expression of HIF-1A in GBC tissues

Immunoreactivity	Nuclear HIF-1A positive	nuclear HIF-1A negative	Total
cytoplasmic HIF-1A positive	15	37	52
cytoplasmic HIF-1A negative	12	63	75
Total	27	100	127

**Table 6:** The expression of HIF-1A in normal tissues

Immunoreactivity	nuclear HIF-1A positive	nuclear HIF-1A negative	Total
cytoplasmic HIF-1A positive	7	24	31
cytoplasmic HIF-1A negative	10	6	16
Total	17	30	47

**Table 7:** Univariate and multivariate analysis of the correlation between clinicopathological parameters and prognostic significance of GBC patients

Variables	Univariate analysis	*p* value	Multivariate analysis	*p* value
	HR (95%CI)		HR(95%CI)	
Gender (male vs. female)	1.316 (0.860-2.013)	0.205		NA
Age (y) (<68 vs. ≥68)	1.265 (0.871-1.837)	0.217		NA
Tumor diameter (cm) (<2 vs. ≥2)	1.424 (0.983-2.063)	0.061		NA
Differentiation (low/undifferentiated vs. high/moderate)	0.566 (0.387-0.827)	**0.003**	0.803 (0.506-1.275)	0.352
Depth of invision (T1/TI vs. T3/T4)	1.683 (1.101-2.572)	**0.016**	1.402 (0.879-2.237)	0.156
Lymph node metastasis (no vs. yes)	1.829 (1.249-2.676)	**0.002**	2.435 (1.078-5.502)	**0.032**
TNM stages (I/II vs. III/IV)	1.653 (1.123-2.433)	**0.011**	0.652 (0.279-1.524)	0.324
Gallstones	1.026 (0.698-1.508)	0.896		NA
AFP (<20 vs. ≥20)	2.107 (0.853-5.207)	0.106		NA
CEA (<5 vs. ≥5)	1.236 (0.790-1.932)	0.353		NA
CA199 (<37 vs. ≥37)	1.198 (0.816-1.758)	0.356		NA
Nuclear HIF-1A expression (positive vs. negative)	2.464 (1.553-3.911)	**< 0.001**	2.129 (1.308-3.466)	**0.002**
Cytoplasmic HIF-1A expression (positive vs. negative)	1.067 (0.730-1.558)	0.738		NA

Note: Variables with *p* values more than 0.05 in the univariate models were not adapted (NA) in the multivariate analysis. *p*<0.05 was defined statistically significant and was given in bold. CI: confidence interval. HR: Hazard ratio.
